# Spatial clustering of overweight/obesity among women in India: Insights from the latest National Family Health Survey

**DOI:** 10.1371/journal.pone.0305205

**Published:** 2024-07-24

**Authors:** Mahashweta Chakrabarty, Subhojit Let

**Affiliations:** Banaras Hindu University, Varanasi, Uttar Pradesh, India; University of Gour Banga, INDIA

## Abstract

**Background:**

Overweight/obesity has become global health concern with increasing prevalence. This study examined district-level disparities and spatial clustering of overweight/obesity among women of reproductive age (WRA) in India using the latest National Family Health Survey-5 (2019–2021) data.

**Method:**

Information of 623,656 women aged 15 to 49 from the NFHS-5 (2019–2021) were analysed in this study. The outcome variable was BMI as classified by the world health organisation (WHO). Utilising Global Moran’s I, Anselin’s Local Moran’s I, and spatial regression models spatial clustering and associated factors were analysed.

**Result:**

The study found that 24% (95% CI: 23.8–24.3) of WRA in India were overweight/obese in 2019–21. The prevalence was greatest in Punjab (41%) and lowest in Meghalaya (11%). Additionally, the Global Moran’s I value for the outcome variable was 0.73, indicating a positive spatial autocorrelation in the overweight/obesity. Districts of Tamil Nadu, Andhra Pradesh, Karnataka, Kerala, Telangana, Punjab, Himachal Pradesh, Jammu & Kashmir, Haryana, and Delhi were hotspots of overweight/obesity. Several factors of overweight/obesity among WRA were identified, including place of residence (β: 0.034, p: 0.011), parity (β: 0.322, p: 0.002), social group (β: -0.031, p: 0.016), religion (β: -0.044, p: <0.001), household wealth status (β: 0.184, p: <0.001), mass-media exposure (β: 0.056, p: 0.031), and diabetes (β: 0.680, p: <0.001).

**Conclusion:**

The study emphasizes the importance of targeted interventions and region-specific strategies, while also stressing the need to address associated factors to develop effective public health initiatives aimed at reducing overweight/obesity prevalence among WRA in India.

## Introduction

Overweight/obesity is a condition marked by the accumulation of excessive body fat [1]. The prevalence of overweight/obesity has witnessed a significant rise worldwide over the past few decades, contributing to a substantial burden of chronic diseases such as cardiovascular diseases, cancer, diabetes, and chronic respiratory diseases [2, 3]. This global trend is concerning, as the prevalence of obesity has tripled since 1975, with over half of the adult population now falling into the overweight/obese category [1]. By 2016, the global prevalence of overweight surpassed 1.9 billion adults, making up nearly 39% of the adult population, while more than 650 million individuals, accounting for 13%, were classified as obese [4]. The projection indicated that by the year 2030, approximately 38% of the global population would be characterized as overweight, with an additional 20% falling into the category of obesity [5].

Overweight/obesity is recognized as major risk factor for multiple non-communicable diseases (NCDs) such as type 2 diabetes, certain types of cancers, musculoskeletal disorders and cardiovascular diseases [6–8]. In 2019, around 5 million deaths from NCDs were linked to having a higher than recommended body mass index (BMI) [9].

In India, there has been a significant shift in the nutritional status among women of reproductive age (WRA) over the years. The prevalence of underweight women has steadily declined from 11.6% in 2000 to 9.7% in 2016. Whereas, there has been a notable increase in overweight/obesity among WRA, rising from 10.6% in 1998–99 to 24.0% in 2019–21 [10–13] Therefore, addressing this issue in India is crucial, especially among WRA as the prevalence not only higher among them but also the fact that overweight/obesity among WRA is associated with multiple health complications, particularly during pregnancy and childbearing, as well as reproductive and sexual health [14, 15].

Prior studies has contributed valuable insights by examining various aspects of overweight/obesity, including socioeconomic differentials in its prevalence among WRAs in India [16, 17]. Additionally, numerous studies have concentrated on investigating the rural-urban gap and have emphasized the influence of various factors on overweight/obesity among WRA [18, 19]. These factors include age, socioeconomic status (SES), educational status, area of residence, and marital status, family size, pregnancy or marital status, and parity, all of which have been associated with overweight/obesity among WRA [20–24].

While previous literature has explored the association between overweight/obesity and various health outcomes, including NCDs, limited attention has been given to investigating the geographical disparities in overweight/obesity prevalence among WRA in India [17, 25]. With significant regional variations in demographic and epidemiological transitions, as well as diverse lifestyle patterns, food habits, socioeconomic development, and healthcare infrastructure across states, the prevalence and determinants of overweight/obesity may differ substantially at both state and district levels [26, 27]. Moreover, it’s important to highlight that earlier research have examined the spatial distribution of overweight/obesity among WRA in India, which are relatively outdated and do not incorporate the latest data, resulting in a significant gap in our comprehension of the current situation regarding overweight/obesity [17, 25, 28]. Given that health is a major responsibility in India, where two-thirds of healthcare spending allocated by state budgets and the rest by the central budget, it’s imperative to study overweight/obesity [29]. Understanding their current geographic distribution at state and district levels, along with associated factors, is crucial.

The present study aims to address this research gap by presenting a comprehensive analysis of district-level disparities and spatial clustering of overweight/obesity among WRA in India, and identifying the socio-economic and biodemographic factors associated with overweight/obesity at the district level. By examining the spatial clustering of overweight/obesity and considering the specific contextual factors influencing overweight/obesity, this study will contribute to a deeper understanding of the relationship between geography, demographics, and overweight/obesity prevalence.

## Methods

### Data source

The data utilized in our study originates from the fifth round of the National Family Health Survey (NFHS-5), which was conducted between 2019 and 2021. The survey aims to gather comprehensive information on population, health, and nutrition across India, including its 28 states, 8 union territories, and 707 districts. A total of 636,699 households were successfully interviewed with a response rate of 97%. The surveys included a participation of 724,115 women aged 15–49 years. Out of 724,115 women, at first, 34,148 were excluded because they were pregnant or had given birth within two months prior to the survey. Additionally, 23,747 women were excluded due to inconsistencies in the BMI values, and 42,564 were excluded because of missing information on independent variables such as social groups and diabetes. Consequently, the final sample size for analysis in this study consisted of 623,656 women aged 15–49 years.

This sample represents women from all 707 districts of India. The details of sampling procedure used in the NFHS and process of data collection are available in the national report which is freely available online [13].

### Ethics approval and consent to participate

This study utilized publicly accessible secondary data. No personally identifiable information about survey respondents is included in the dataset. This study only used anonymous public-use dataset provided by the Demographic and Health Surveys (DHS) program, therefore, no ethical approval is required for this study. The survey data used in this study can be obtained by making a formal request on the official website of the DHS program https://dhsprogram.com/data/available-datasets.cfm.

### Outcome variable

The primary focus of our study is the BMI, which serves as the outcome variable. BMI is a widely used measure that assesses an individual’s weight in relation to their height, providing insights into their nutritional status. The calculation of BMI involves dividing a person’s weight in kilograms by the square of their height in meters (kg/m^2^). This computation yields the BMI value [30].

To facilitate the interpretation and analysis of BMI data, the WHO has established four broad categories based on BMI values: (1) underweight (BMI < 18.5 kg/m^2^), (2) normal weight (BMI 18.5–24.9 kg/m^2^), (3) overweight (BMI 25.0–29.9 kg/m^2^), and (4) obese (BMI ≥ 30.0 kg/m^2^). In our study, we have adhered to the WHO’s BMI cut-off values.

In order to transform the continuous BMI variable into a binary outcome variable for our analysis, we have coded it as follows: a value of 1 is assigned if the BMI is ≥ 25.0 kg/m^2^, indicating woman is overweight/obese, while a value of 0 is assigned if the BMI is ≤ 24.9 kg/m^2^, indicating that woman is not overweight/obese.

### Independent variables

The spatial analysis in our study included various independent variables: age of respondents (in years), place of residence (urban, rural), marital status (not married, currently married), parity (one or more children, no child), level of education (higher and above, below higher), social group (Scheduled Caste (SC)/Scheduled Tribe (ST), non-SC/ST), religion (Hindu, non-Hindu), household wealth (non-poor, poor), mass-media exposure (at least one media exposure, no mass-media exposure), consumption of fried food and aerated drinks (yes, no), alcohol consumption (yes, no), consumption of tobacco (yes, no), and currently having diabetes (yes, no). These variables were chosen based on their potential influence on the outcome variable and were utilized to explore their associations within the spatial context of the study [16, 28, 31, 32].

### Statistical analysis

In our study investigating the spatial heterogeneity in overweight/obesity among WRA in India, we employed a robust statistical analysis to comprehensively analyse the geographical differences.

To assess the spatial distribution of overweight/obesity prevalence, we computed the proportion of WRA with overweight/obesity at both the state and district levels. This involved dividing the total number of women with overweight/obesity in each district by the total number of women residing in that district. By representing these proportions on a district-level map, we were able to visually depict the spatial patterns and distribution of overweight/obesity prevalence across India’s states and districts.

#### Detection of spatial autocorrelation

While visual identification of clustered and non-clustered districts based on prevalence maps can offer preliminary insights, it is imperative to employ rigorous statistical tests to determine the presence and statistical significance of clustering. To this end, we employed the widely used Moran’s I test, which is a spatial autocorrelation test. This test allows us to examine whether districts with similar or dissimilar overweight/obesity prevalence values exhibit clustering. Moran’s I statistic quantifies the correlation coefficient between the overweight/obesity prevalence in a specific district and the values of the same variable in surrounding districts, thereby capturing the spatial autocorrelation.

#### Global Moran’s I test

To ascertain the magnitude and significance of spatial autocorrelation, we utilized the Global Moran’s I statistic. This measure provides an overview of the overall spatial clustering or autocorrelation in the data. The Global Moran’s I range from -1 to +1, where values close to -1 indicate perfect clustering of dissimilar values, values close to +1 indicate perfect clustering of similar values, and a value of 0 denotes a complete spatial randomness in the distribution of overweight/obesity prevalence.

#### Cluster and outlier analysis (Anselin’s Local Moran’s I)

To pinpoint statistically significant clusters and outliers with high or low overweight/obesity prevalence, we conducted a comprehensive cluster and outlier analysis utilizing Anselin’s Local Moran’s I (Anselin, 1995, 2020). This analysis enabled the identification of significant hotspots (high-high clusters) and cold spots (low-low clusters) across districts, providing insights into the geographical concentration of overweight/obesity prevalence. Additionally, the analysis detected high-low outliers and low-high outliers, indicating districts with values that deviated from their neighbouring areas. To determine spatial contiguity, we employed Queen Contiguity weights, considering both shared edges and corners between geographical units [33].

#### Spatial regression models

Furthermore, to explore the factors associated with overweight/obesity among WRA in India, we conducted spatial regression analysis at the district level. The district served as the primary unit of analysis, while the proportion of women with overweight/obesity represented the outcome variable. Initially, we employed ordinary least squares (OLS) regression, and to account for potential spatial dependence in the dataset, we conducted diagnostic tests such as the lagrange multiplier (LM) and robust lagrange multiplier (RLM) tests [34]. The detection of significant results from these tests suggested the presence of spatial dependence, prompting us to compare the spatial lag model (SLM) and spatial error model (SEM) to determine the most suitable fit for our data. The selection process involved considering various criteria, including the akaike information criterion (AIC), log-likelihood values, LM, and Robust LM values [35]. Ultimately, based on the model fit criteria, we identified the SEM as the preferred model due to its lowest AIC value and highest log-likelihood, LM, and Robust LM values [34].

For conducting these rigorous statistical analyses, we employed reputable software packages such as ArcGIS 10.5, GeoDa, GeoDaSpace, and Stata 16 [36–38].

## Results

### Respondents’ characteristics

Table 1 presents the socio-demographic characteristics of WRA in NFHS-5. Approximately one-third of these women fell within the 20–29 age bracket. The majority, accounting for nearly 70%, resided in rural areas, while over 70% were presently married. In terms of parity, more than 40% of women had 1–2 children. Moreover, over 80% of women had educational attainment below the higher education level. Additionally, nearly 70% of the women identified as non-SC/ST. As for dietary habits, more than 80% of women reported consuming fried foods and aerated drinks. Conversely, minimal consumption of alcohol and tobacco, as well as low prevalence of diabetes, were observed.

**Table 1:** Respondent characteristics by background characteristics, NFHS-5, 2019–2021.

Background characteristics	NFHS 5	
	Frequency (N = 623,656)	%^a^
**Age (in years)**		
15–19	106,570	17.1
20–29	191,767	30.8
30–39	174,641	28.0
40–49	150,678	24.2
**Place of residence**		
Rural	425,270	68.2
Urban	198,386	31.8
**Marital status**		
Currently married	442,296	70.9
Not married	153,722	24.7
Formerly married	27,637	4.4
**Parity**		
No child	189,202	30.3
1–2 children	253,067	40.6
3–4 children	143,644	23.0
5 and above	37,743	6.1
**Level of education**		
Below higher	527,885	84.6
Higher	95,771	15.4
**Social groups**		
Non-SC/ST	417,523	67.0
SC/ST	206,133	33.1
**Religion**		
Hindu	523,369	83.9
Muslim	68,572	11.0
Christian	14,611	2.3
Others	17,104	2.7
**Household wealth**		
Poorest	112,398	18.0
Poorer	124,943	20.0
Middle	130,974	21.0
Richer	131,715	21.1
Richest	123,626	19.8
**Mass media exposure**		
No mass media exposure	136,829	21.9
At least one mass media exposure	486,827	78.1
**Eat fried food and aerated drink**		
No	108,883	17.5
Yes	514,773	82.5
**Alcohol consumption**		
No	618,814	99.2
Yes	4,842	0.8
**Consumption of tobacco in any form**		
No tobacco	598,508	96.0
Uses tobacco: smoke or smokeless	25,148	4.0
**Currently having diabetes**		
No	611,771	98.1
Yes	11,885	1.9

Note: N: sample; SC: Scheduled Caste; ST: Scheduled Tribe

^***a***^: all percentages are weighted.

### Spatial patterns of overweight/obesity among WRA across 28 states, 8 UTs, and 707 districts of India

**Fig 1:**
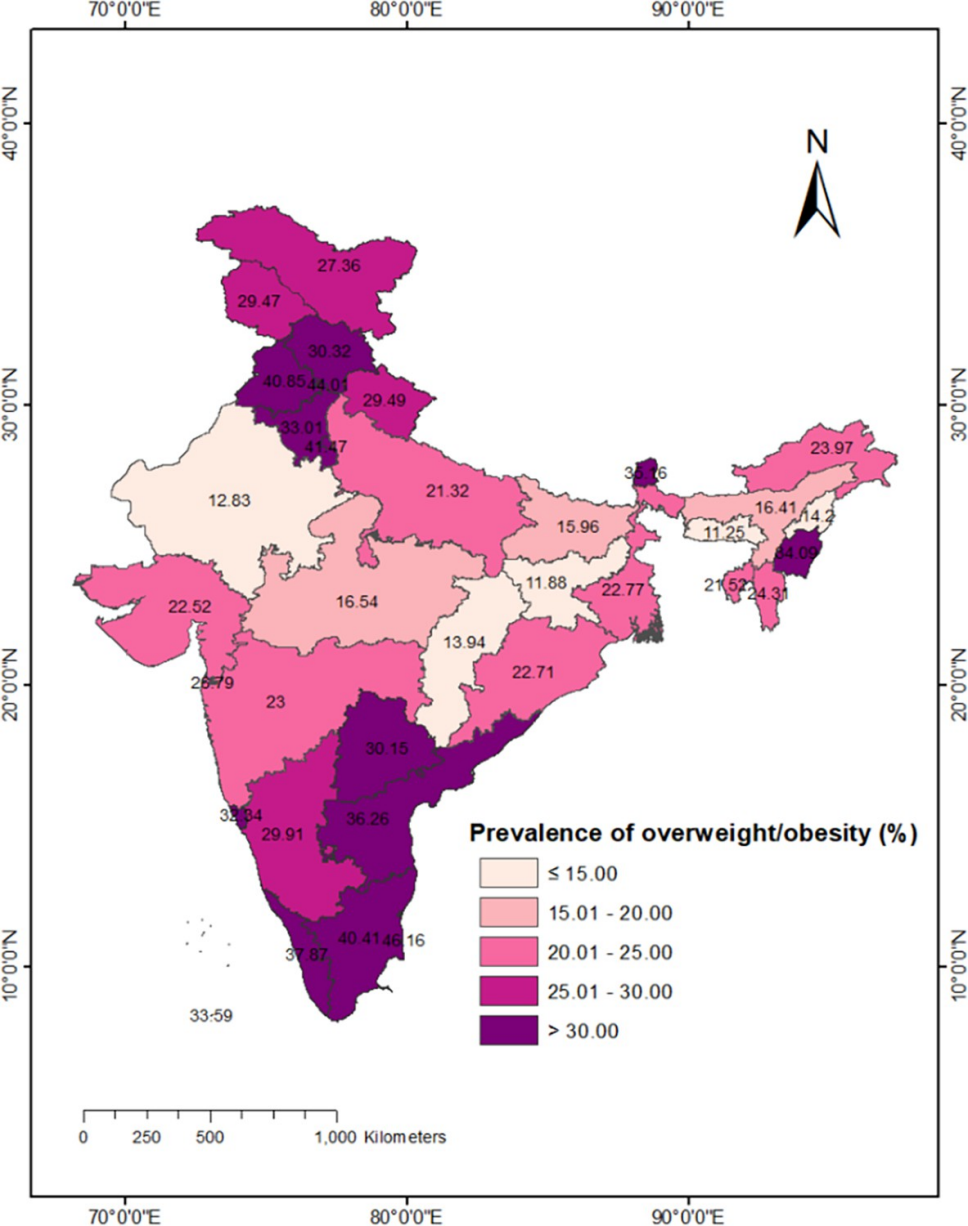
Overweight/obesity among WRA across the states of India, NFHS-5, 2019–21. As far as the base layer is concerned, we used a shapefile file freely downloadable from Source: This map is authors’ own creations. https://spatialdata.dhsprogram.com/boundaries/#view=table&amp%3BcountryId=IA&countryId=AF for national and sub-national boundaries.

Our study findings highlight the significant variation in overweight/obesity among WRA in India during the period of 2019–21. Overall, approximately 24% of WRA were found to be overweight/obese in India. However, this percentage exhibited substantial spatial heterogeneity across different states in India. Fig 1 provides a visual representation of the state-level variation in overweight/obesity prevalence among WRA. Notably, the prevalence reached as high as 41% in Punjab, while it was considerably lower at 11% in Meghalaya. This wide range of prevalence indicates an unequal burden of overweight/obesity across the geographical regions of India.

Fig 1 revealed that a total of ten states exhibited the highest prevalence of overweight/obesity, surpassing 30%. These states include Punjab, Tamil Nadu, Kerala, Andhra Pradesh, Sikkim, Manipur, Haryana, Goa, Himachal Pradesh, and Telangana. Among the Union Territories (UTs), the highest prevalence rates were observed in Puducherry (46%), Chandigarh (44%), and Delhi (41%).

**Fig 2:**
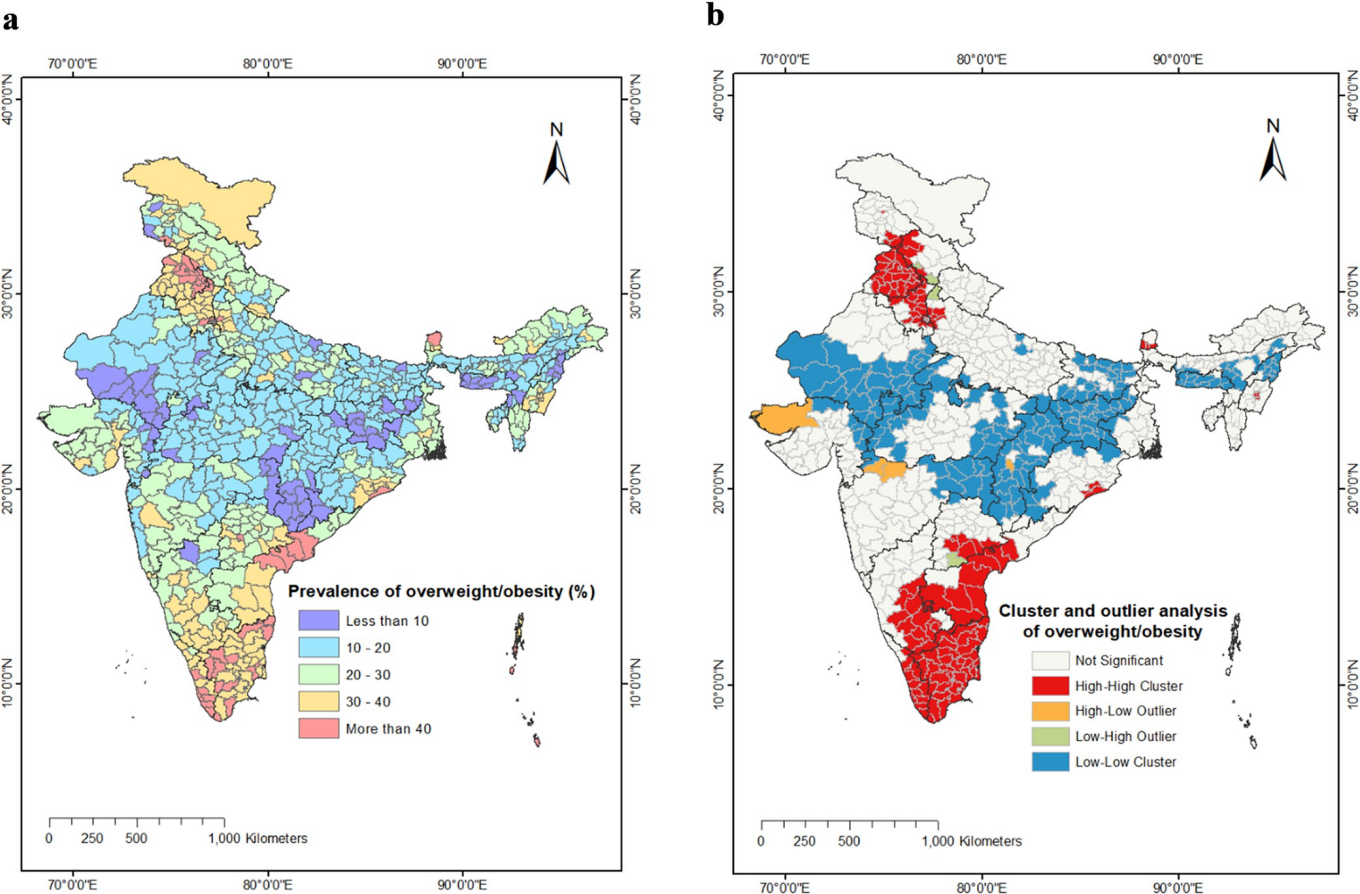
a) District-wise map showing prevalence of overweight/obesity among WRA in India; NFHS-5, 2019–21 b) Cluster and outlier analysis map (Anselin Local Moran’s I) showing the statistically significant (p value: <0.05) spatial clusters and outliers of overweight/obesity among WRA across the districts of India, NFHS-5, 2019–21. Source: All the maps used are the authors’ own creations. As far as the base layer is concerned, we used a shapefile file freely downloadable from https://spatialdata.dhsprogram.com/boundaries/#view=table&amp%3BcountryId=IA&countryId=AF for national and sub-national boundaries.

However, the state-wise distribution masked the substantial variation within each state, that is why we have dived deeper into district-level variation in overweight/obesity in WRA Fig 2A, depicting the district-level distribution of overweight/obesity among WRA, revealed a significantly more diverse pattern compared to the state-level analysis. The prevalence of overweight/obesity ranged from 54% in the Kanyakumari district of Tamil Nadu to 4% in the Balaghat district of Madhya Pradesh.

Within the southern states, notably high prevalence rates of overweight/obesity were observed in multiple districts. These included Kanniyakumari, Coimbatore, Thiruvallur, Kancheepuram, Vellore, Tiruppur, Namakkal, Chennai, Theni, Thanjavur, Thoothukkudi, Madurai, and Erode districts of Tamil Nadu; Thiruvananthapuram, Kollam, Pathanamthitta, Thrissur, Alappuzha, and Kottayam districts of Kerala; Guntur, West Godavari, East Godavari, and Krishna districts of Andhra Pradesh; as well as Hyderabad and Medchal-Malkajgiri districts of Telangana.

Furthermore, among the northern Indian states, high prevalence rates exceeding 40% were evident in Sahibzada Ajit Singh, Jalandhar, Fatehgarh Sahib, Ludhiana, Rupnagar, Patiala, Kapurthala, Shahid Bhagat Singh Nagar, Amritsar, and Hoshiarpur districts of Punjab; as well as Jhajjar, Ambala, and Panchkula districts of Haryana.

In contrast, several districts in southern Rajasthan, Chhattisgarh, Odisha, Jharkhand, and the eastern districts of Gujarat and Maharashtra exhibited considerably lower prevalence rates of overweight/obesity, generally below 10%.

A simple visual examination of the overweight/obesity map does not provide insights into whether overweight/obesity is spatially clustered, randomly distributed, or dispersed across the districts of India. To investigate the spatial pattern, we conducted a Global Moran’s I analysis using the districts of India as feature locations and the corresponding prevalence of overweight/obesity. The results of the spatial autocorrelation analysis demonstrated a significant clustering pattern in the spatial distribution of overweight/obesity, with a Moran’s index of 0.73 (p-value < 0.001) (see S1 File for detailed report). The associated Z-score was determined to be 31.17, indicating that the observed clustered pattern in the overweight/obesity prevalence map had less than a 1% probability of occurring by random chance alone. These findings confirm the presence of a spatial dependency and highlight the need to consider spatial factors in understanding and addressing overweight/obesity at the district level in India.

In Fig 2B, we present the cluster and outlier map depicting the spatial distribution of overweight/obesity. The colour scheme used in the map helps identify different patterns: red represents high-high clusters, blue represents low-low clusters, orange indicates high-low outliers, and light green indicates low-high outliers.

The presence of high-high clusters, also known as hot-spots, indicates districts with above-average prevalence of overweight/obesity that are geographically adjacent to other districts with similarly high prevalence. Our analysis revealed two major regions in India where statistically significant hot-spots were identified. The first region encompasses the southern Indian states, specifically all districts in Tamil Nadu, Andhra Pradesh (excluding Kurnool district), and certain southern districts of Telangana. Additionally, all districts in Kerala, except for a few in the northern part, exhibited significant hot-spots. The second region with significant hot-spots was observed in north India, particularly in the districts of Punjab and Haryana.

In contrast, districts in Chhattisgarh, Jharkhand, eastern Maharashtra, eastern and western Madhya Pradesh, and districts of Rajasthan, excluding the northern part, showed a different pattern. These regions exhibited low prevalence of overweight/obesity, forming low-low clusters.

Furthermore, the map also identified high-low outliers, indicating districts with above-average prevalence surrounded by districts with below-average prevalence. Notably, Kachchh district in Gujarat, as well as Dhule and Jalgaon districts in Maharashtra, displayed this pattern.

### Results of spatial regression

To identify the relevant determinants of overweight/obesity, we employed three regression models: OLS, SLM, and SEM. Table 2 presents the results obtained from these models. Initially, OLS estimation was performed to examine the association between overweight/obesity and its correlates without considering the spatial structure of the data. Two sets of LM and robust LM tests were conducted during OLS estimation to assess the suitability of the model in predicting spatial dependence in our dataset. Both SLM and SEM were subsequently employed in the analysis, as the LM and robust LM values for SLM and SEM indicated statistically significant spatial dependence in the dataset. Notably, the robust LM value for the error term exceeded the robust LM value for the lag term. Furthermore, SEM exhibited the lowest AIC and SW values, as well as the highest log likelihood value, indicating a better fit and suitability of the model. Consequently, SEM was selected as the preferred model to investigate the spatial dependence of overweight/obesity among WRA.

**Table 2:** Spatial regression model for estimating spatial association between percentage of overweight/obesity among women (15–49 years) and background characteristics, NFHS-5, 2019–2021.

Variables	OLS	SLM	SEM
**Age of respondents (in years)**	-0.002 (0.335)	0.000 (0.830)	-0.002 (0.344)
**Place of residence (urban)**	0.014 (0.357)	0.036 (0.004)	0.034 (0.011)
**Marital status (not married)**	-0.323 (0.007)	-0.080 (0.415)	-0.016 (0.872)
**Parity (one or more children)**	0.575 (<0.001)	0.264 (0.006)	0.322 (0.002)
**Level of education (higher and above)**	0.351 (<0.001)	0.176 (<0.001)	0.096 (0.062)
**SC/ST**	-0.063 (<0.001)	-0.040 (0.001)	-0.031 (0.016)
**Hindu**	-0.059 (<0.001)	-0.043 (<0.001)	-0.044 (<0.001)
**Non-poor**	0.160 (<0.001)	0.096 (<0.001)	0.184 (<0.001)
**Mass media exposure (at least one media exposure)**	0.050 (0.095)	0.034 (0.162)	0.056 (0.031)
**Eat fried food and aerated drink**	0.052 (0.034)	0.055 (0.006)	-0.029 (0.202)
**Alcohol consumption (yes)**	0.193 (<0.001)	0.092 (0.034)	0.030 (0.587)
**Consumption of tobacco (yes)**	-0.009 (0.742)	0.020 (0.390)	0.023 (0.490)
**Currently having diabetes (yes)**	1.669 (<0.001)	1.055 (<0.001)	0.680 (<0.001)
**Rho**		0.478	
**Lambda**			0.815
**AIC**	-1975.05	-2224.11	-2382.41
**SW**	-1911.16	-2155.65	-2318.52
**Log Likelihood**	1001.53	1127.05	1205.21

Note: OLS: Ordinary least squares; SLM: Spatial lag model; SEM: Spatial error model; AIC: Akaike information criterion; SW: Schwarz criterion.

The results of the SEM are presented in Table 2. The lambda value of 0.81 (p < 0.001) indicates a highly significant spatial dependence of the outcome variable on neighbouring values. The SEM analysis confirmed several statistically significant predictors of overweight/obesity among WRA.

According to the SEM, the following variables were found to be significant predictors of overweight/obesity among WRA: place of residence (β: 0.034, p: 0.011), parity (β: 0.322, p: 0.002), social group (β: -0.031, p: 0.016), religion (β: -0.044, p: <0.001), household wealth status (β: 0.184, p: <0.001), mass-media exposure (β: 0.056, p: 0.031), and diabetes (β: 0.680, p: <0.001).

To elaborate, the SEM indicates that a 10% increase in the proportion of women with one or more children is associated with a significant 3.2% decrease in overweight/obesity. Furthermore, non-poor women are at a 1.8% higher risk of overweight/obesity compared to those from poorer households.

In summary, the SEM highlights that urban residence, having one or more children, non-poor household wealth, mass-media exposure, and diabetes are positively associated with overweight/obesity among WRA. Conversely, belonging to the SC/ST community and being Hindu exhibit a negative association with overweight/obesity among WRA.

## Discussion

The main objective of the present study was to investigate district-level disparities and clustering of overweight/obesity among WRA in India. Specifically, we aimed to identify clusters of overweight/obesity, focusing on cold-spots and hot-spots at the district level. Additionally, the study sought to examine the association between overweight/obesity among WRA and various socioeconomic and biodemographic factors at the district level.

The results of our study revealed that, at the national level during 2019–21, around 24% of WRA had overweight/obesity, indicating a significant public health concern. Substantial geographic disparity was observed at the state level, with Punjab having the highest percentage of WRA with overweight/obesity and Meghalaya the lowest. Our district-level analysis revealed even more diverse variations in overweight/obesity compared to the state-level analysis. Notably, we identified hot-spots with significantly higher prevalence in the southern states of Tamil Nadu, Andhra Pradesh, Telangana, and Kerala, as well as in Punjab and Haryana in North India. Conversely, districts in Chhattisgarh, Jharkhand, eastern Maharashtra, eastern and western Madhya Pradesh, Rajasthan (excluding the northern part), and the northeastern states exhibited lower prevalence.

Overall, the findings indicated a clear spatial clustering pattern in the distribution of overweight/obesity, with districts exhibiting similar prevalence levels being geographically clustered. This clustering pattern suggests the presence of localized factors contributing to the high prevalence of overweight/obesity in specific regions. In terms of the factors associated with overweight/obesity among WRA, the study identified several significant predictors. Urban residence, having one or more children, non-poor household wealth, mass-media exposure, and diabetes were positively associated with overweight/obesity [39, 40]. Conversely, belonging to the SC/ST community and being Hindu showed a negative association. These findings are also supported by previous researches on overweight/obesity [41, 42].

The positive association between urban residence and overweight/obesity among WRA can be attributed to various factors. Urban areas often provide greater access to processed and high-calorie foods, which can contribute to weight gain [43]. Additionally, urban environments may promote sedentary lifestyles and reduce opportunities for physical activity [44, 45]. These factors, coupled with the potential influence of socio-cultural changes and urbanization on dietary patterns, may explain the higher prevalence of overweight/obesity in urban settings [46].

The association between having one or more children and overweight/obesity among WRA can be explained by multiple factors [21]. Pregnancy and childbirth can lead to changes in body composition and hormonal imbalances, which may contribute to weight gain and difficulty in losing excess weight postpartum [47, 48]. Non-poor household wealth has been consistently associated with a higher risk of overweight/obesity among WRA. Higher income levels often provide greater purchasing power, allowing individuals to afford energy-dense foods and engage in sedentary behaviours [49, 50]. Additionally, socio-economic disparities may influence access to resources and opportunities for physical activity, resulting in differential obesity rates among different wealth groups [51].

Mass-media exposure has been linked to increased consumption of high-calorie foods, and sedentary behaviours, which can contribute to overweight/obesity [52]. The way the media shows ideal body images and promotes unhealthy eating habits can affect how people see themselves and manage their weight [53]. This connection highlights the importance of promoting media literacy and delivering health-focused messages in the context of overweight/obesity prevention.

The positive association between diabetes and overweight/obesity is well-established. Insulin resistance, a characteristic of type 2 diabetes, can lead to weight gain and contribute to the development of overweight/obesity [6–8]. The bidirectional relationship between diabetes and overweight/obesity further complicates the issue, as excess weight can increase the risk of developing diabetes, while diabetes itself can contribute to weight gain [54]. On the other hand, the negative association between belonging to the SC/ST community and being Hindu with overweight/obesity among WRA may be influenced by various cultural and socio-economic factors. Previous studies have suggested that cultural practices, dietary preferences, and lifestyle habits within specific communities can influence body weight and adiposity [45, 46]. Socio-economic factors and access to healthcare facilities may also contribute to the observed disparities in overweight/obesity prevalence among different socio-religious groups.

Overall, these findings underscore the need for targeted interventions and policies that address the district-level disparities and clustering of overweight/obesity among WRA in India. Efforts should focus on implementing region-specific strategies that consider the socio-demographic and cultural factors influencing overweight/obesity prevalence. By targeting high-prevalence areas and addressing the identified determinants, effective measures can be developed to combat overweight/obesity and improve the overall health and well-being of WRA in India.

### Strengths and limitations

The study demonstrates several notable strengths. Firstly, using latest dataset it identifies significant clusters of overweight/obesity among WRA in India, which provides the latest picture of this issue across the Indian districts. Secondly, the study’s large sample size enhances the generalizability and validity of its findings. By using spatial regression analysis, the study effectively addresses spatial dependency, further strengthening the reliability of the results.

However, certain limitations should be acknowledged. Firstly, the cross-sectional study design restricts the ability to establish causal relationships between variables. Furthermore, categorizing all women with a BMI ≤24.9 kg/m^2^ as not overweight/obese inadvertently includes underweight women (BMI <18.5 kg/m^2^). This categorization could influence the Anselin’s local Moran’s I results, particularly in regions with a high prevalence of underweight women. Additionally, the absence of important covariate data, such as physical activity patterns, dietary habits, and family history, may have introduced confounding biases in the analysis. Furthermore, while the BMI is an important indicator recommended by the WHO for measuring nutritional status, it should be noted that categorizing individuals solely based on BMI may result in misclassification of individual data. BMI does not differentiate between body fat and lean body mass, whereas measurements such as waist circumference and waist-to-hip ratio provide a better reflection of abdominal obesity, which is a significant risk factor for various health conditions.

## Conclusion

This study revealed significant district-level disparities and clustering of overweight/obesity among WRA in India, with a national prevalence of approximately 24%, highlighting a major public health concern. Spatial analysis identified distinct hot-spots and cold-spots, with higher prevalence in southern states and certain northern regions. Factors such as urban residence, having children, higher household wealth, mass-media exposure, and diabetes were positively associated with overweight/obesity, while belonging to the SC/ST community and being Hindu were negatively associated. These findings underscore the importance of targeted interventions and region-specific strategies to address these disparities and improve the health and well-being of WRA in India.

## Supporting information

S1 File Spatial autocorrelation report (Global Moran*’*s I). (DOCX)
