# Supplementary figures and images for: Spatial clustering of overweight/obesity among women in India: Insights from the latest National Family Health Survey
Source: PLoS One. 2024 Jul 24;19(7):e0305205. doi: 10.1371/journal.pone.0305205 (PMC11268665; doi:10.1371/journal.pone.0305205)

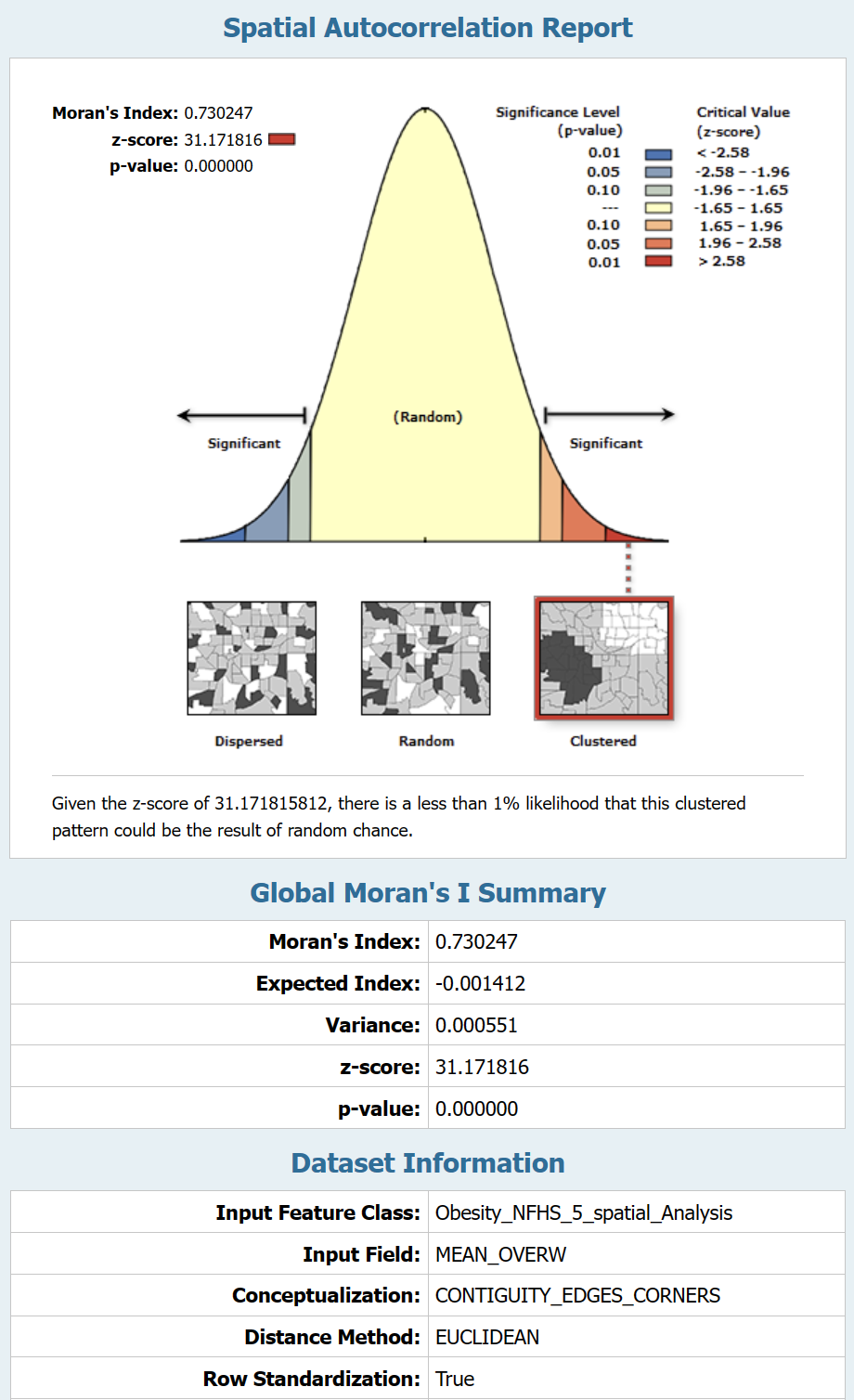


**Supplementary file 1: Spatial autocorrelation report (Global Moran’s I)**

Supplement: S1 File — (DOCX) [file pone.0305205.s001.docx]
